# Micro Change Agents for Gender Equality: Transforming European Research Performing Organizations

**DOI:** 10.3389/fsoc.2021.741886

**Published:** 2021-10-11

**Authors:** Jennifer Dahmen-Adkins, Helen Peterson

**Affiliations:** ^1^ Institute of Sociology, RWTH Aachen University, Aachen, Germany; ^2^ School of Humanities, Education and Social Sciences, University of Örebro, Örebro, Sweden

**Keywords:** gender equality, change agents, action reseach, gender equality plans, implementation theory, transformative science, monitoring, structural change project

## Abstract

This article explores the experiences of micro change agents for gender equality in seven European Research Performing Organizations in seven different countries. The micro change agents were all participants of an international collaborative project consortium, implementing gender equality plans (GEPs), and funded by the European Commission during 4 years. The analysis draws on empirical data consisting of information submitted by the micro change agents during these 4 years and collected using three different monitoring tools, developed within the project to follow the progress of the implementation efforts, but also to provide an arena for individual and collaborative reflection and knowledge exchange between the partners. The aim of the article is to present a systematic analysis of the change practices that these micro change agents experienced as useful and important for promoting gender equality in their different organizational contexts. A total of six such micro change practices are identified, emerging from the empirical data: 1. communicating, 2. community building, 3. building trust and legitimacy, 4. accumulating and using resources, 5. using and transferring knowledge, and 6. drawing on personal motivation. The findings illustrate the multifaceted character of micro change agency for gender equality, particularly in a time-limited project context with a designated funding period. The results from this study can be useful when developing gender equality strategies, policies and practices and can also be used to empower gender equality micro change agents that face challenges while trying to implement GEPs and promote structural change in any kind of institution.

## Introduction

Research within the field of critical gender equality studies has highlighted the importance of macro change agents for gender equality, i.e., organizational leaders and managers with formal, positional power who promote gender equality in their institution (cf. e.g., Peterson 2014; Kelan and Wratil 2018; O’Connor et al., 2019). There is no denying these macro change agents have an important role in achieving sustainable, structural change in any organization, including Research Performing Organizations (RPOs). Notwithstanding, this paper shifts the focus from these macro change agents to the so-called micro change agents, i.e., those who try to change their organization from within, sometimes referred to as “tempered radicals” (Meyerson and Scully 1995; Meyerson and Tompkins 2007). Although previous research has produced valuable accounts about the challenges and successes of micro change agents, we still know less about the change practices they adopt and use, and how these vary depending on setting and how they develop and are modified throughout the duration of a change project.

To add to the already existing literature on change agents for gender equality this article explores the experiences of micro change agents for gender equality in seven RPOs in seven European countries. It adopts a qualitative methodology and a theoretical framework inspired by implementation theory and action research. Despite the interest in the change agent role, there is still a lack of studies applying a theoretically informed approach to micro change agents and their experiences. The aim of the paper is therefore to systematize micro change agents’ experiences of change agency, focusing specifically on their experiences of key change practices. The analysis adopts a practice-based approach to change agency (cf. Caldwell 2012) meaning that it is based on how change agents themselves experience and describe the actual work of practicing change agency in the day-to-day work. Practices are here understood and defined as: “embodied, materially mediated arrays of human activity centrally organized around shared practical understanding” (Schatzki 2005, 11).

The purpose of the article is therefore to explore how change agency is experienced by actors tasked with promoting and enacting gender equality in their organizations. The paper thus addresses the following main research question:

Which change practices do the micro change agents use to promote and enact change towards increased gender equality in their organization, even when their resources might be limited or restricted?

The paper continues with a brief background that introduces the specific context in which the micro change agents in this study are embedded. After that follows two sections where the previous research and the theoretical framework is introduced. This is followed by a section which describes the methodology and method adopted to produce the empirical data analysed in the paper. The subsequent section introduces the results and the analyses. The paper ends with a discussion and conclusion.

## Background

Since 2010, the European Union has made significant efforts to promote structural change in RPOs with the aim to overcome institutional barriers that hinder these institutions in achieving gender equality (European Commission 2012). Gender equality plans (GEPs) serve as a means of accomplishing this objective. Since 2014 institutions participating in projects funded within the Horizon 2020 Framework Programme (H2020) have drafted GEPs to be implemented during the project period. The content of the GEPs is oriented towards the three gender equality goals for fostering institutional change defined for the European Research Area: 1. removing barriers to the recruitment, retention and career progression of female researchers, 2. working towards a fair gender balance in decision-making processes and bodies, and 3. taking the gender dimension into account in the content of research and innovation (European Commission 2020). Speaking in numbers, until 2020 18 GEP consortia received funding in H2020, which translates into a budget of 43.9 € million for 168 participating institutions of which 130 RPOs are involved as partners implementing GEPs. The remaining beneficiaries are either involved in an evaluating, technical or consulting role (ibid, 22). For all funded projects, accompanying monitoring and evaluation is mandatory in order to identify successful institutional strategies and gender equality measures and make them transferable to other stakeholders. This process also enables the identification of structural and individual challenges, as well as the discussion of experienced resistance. The micro change agents, whose experiences are analyzed in this article, were active within one of these structural change projects funded in the predecessor program of H2020, the 7th Framework Programme of the EU Commission. The project aimed at identifying and implementing the best systemic approaches to increase the participation and career advancement of women researchers through the implementation of a tailor-made GEP in seven European RPOs in seven European countries.

Recently, the European Commission went one step further and announced that GEPs will be an eligibility criterion for public institutions in EU Member States and Associated Countries seeking funding under the new Horizon Europe Framework Programme from 2022 onwards. In order to be recognized, institutional GEPs must fulfil certain requirements, including the publication of the document signed by top management, the presentation of specific resources and expertise in the field of gender equality, the collection and annual reporting of sex/gender-disaggregated data, and finally the provision of gender equality training for staff and management (European Commission 2021). These new political developments are now forcing public RPOs that have neglected or not systematically focused on gender equality as an institutional task to address this issue if they want to successfully apply for funding. As a result, a new generation of micro change agents will evolve who will have to face the challenges of structural change.

## Previous Research on Change Agency

### Change Agents for Gender Equality

Change agents are actors who facilitate, promote, coordinate, champion and implement change in organizations (Caldwell 2006). They can play an important role when it comes to changing organizations towards increased gender equality (Meyerson and Tompkins 2007). Previous research has pointed out primarily three key factors as essential for efficient change agency within the field of gender equality. First of all, change agents need to be willing to take on the role as change agents (Parsons and Priola 2013). Second, change agents need awareness of gendering processes in organizations. A sensitivity to gender inequalities have been described as an important prerequisite for change agents for gender equality (Peterson 2014). Previous research suggests that this kind of awareness can develop through direct experiences of being marginalized, which implies that women are more motivated to initiate change than men (Meyerson and Tompkins 2007).

Many women with the awareness and will to act as change agents, however, are in practice restricted by their marginalized position in organizations, which leaves them with a lack of power, influence and resources necessary to initiate change (Wroblewski 2019). Consequently, awareness is in itself insufficient to produce effective change agents for gender equality (Parsons and Priola 2013). Therefore, the third key factor, necessary for change agency, is the authority to disrupt and challenge those organizational routines and practices that reproduce gender imbalance and inequalities (Linstead et al., 2005). Senior managers possess the authority to set strategic goals and implement them, and their commitment has proven to be important in facilitating change and engaging employees to also commit to change (McRoy and Gibbs 2009; Kelan and Wratil 2018). Women managers in further and higher education have for example displayed managerial practices infused by gender awareness and change agency to reduce the impact of the glass ceiling (Deem et al., 2000; Mavin and Bryans 2002; Neale 2011; Cook and Glass 2014).

### Micro and Macro Change Agents

This article makes the distinction between so-called “micro” and “macro” change agents, as defined by Kelan and Wratil (2018). Macro change agents are CEOs and other top-level managers and leaders committed to “drive change toward gender equality, diversity and inclusion in their organisations” (Kelan and Wratil 2018, 6). They use change practices characterized by being in control and taking charge. In contrast, micro change agents use tools and tactics to change their organizations from within, and are doing so from “their individual sphere of influence” (Kelan and Wratil 2018, 6).

Kelan and Wratil (2018) equals micro change agents with so-called “tempered radicals,” a concept first used by Meyerson and Scully (1995), referring to individuals who are committed both to their workplaces and to an ideology or to a cause that is at odds with the dominant culture at work, motivating them to wanting to change the status quo in their organization. Tempered radicals have a marginalized position in their organization and lack authority, legitimacy and resources to mobilize change and therefore need to: “rely on incremental and subversive change tactics that range from subtle, identity-based moves to small, isolated acts to grass-roots coalition building” (Meyerson & Tompkins 2007, 310). We do not adopt the definition of tempered radicals throughout this article because the micro change agents in our study did not all share all of these circumstances and characteristics. This article, however, focuses on micro change agents as insiders (Ackers 2000), who are trying to change the organization that they themselves belong to, but who are also tied to, and committed to a community of other change agents in other organizations, via a structural change project. Certain aspects of the tempered radicals thus applied to several of the micro change agents, as the analysis below will highlight.

### Challenges for Change Agency

The implementation of gender equality change in organizations commonly encounters manifestations of resistance and the reasons for this are complex and multidimensional (Benschop and van den Brink 2014). Gendered organizational structures and practices and masculine cultures are notoriously persistent, rigid and resistant to change (Acker 2000; Thomas and Davies 2005). Gender equality change challenges norms, practices and assumptions regarding the relationships between women and men, but also calls into question personal identities and beliefs (Lombardo and Mergaert 2013). Change efforts also necessarily threaten existing power structures and relationships built on privileges and dominance of certain groups (cf. e.g., Linstead et al., 2005). Micro change agency is thus a difficult task and change agents for gender equality often face both explicit and implicit resistance (Lombardo and Mergaert 2013).

It is therefore of interest to understand which practices micro change agents and tempered radicals use to promote their causes, to receive resources for their causes and/or build a collective movement in their organization. Kelan and Wratil (2018) identify six such change practices for macro change agents who want to drive change toward gender equality, diversity and inclusion: communicating, building ownership, creating accountability, spearheading initiatives, leading by example, and driving culture change. This article similarly identifies six practices for micro change agents who want to drive change toward gender equality in their organizations.

## Theoretical Framework

### Action Research

The study that this article draws on was informed by an action research approach and the analysis of the empirical data is produced within the framework of some of the key concepts of this approach. The purpose of undertaking action research is to bring about change in a specific context. It is a method used for improving practice and implementing changes in practice. An action research project demands careful planning and researchers that can generate solutions to practical problems and involve practitioners in the implementation and development activities (McNiff and Whitehead 2005). Evaluation, monitoring and critical reflection on the process and the outcomes of change is essential (Coleman and Ripping 2000). Action research also generates knowledge based on systematic enquiries and observations conducted within specific and practical contexts. Knowledge is produced when the researchers and participants reflect on processes of change and obtain greater and enhanced understanding, which can lead to revision of initial plans for action (Reason and Bradbury 2008). The character of the knowledge is very specific as it can be used to inform practical application and solutions to specific problems.

Coleman and Ripping (2000) emphasize the importance of working collaboratively in organizational change projects and develop a specific collaborative action research approach to gender organizational change. They involved people inside of the organization they studied as organizational co-researchers through a process of collaborative inquiry which aims to interrupt the power dynamics of research on people and instead focus research with people. Involving people inside of the organization also has the benefit of increasing the chances that changes implemented will be long-term and sustainable by strengthening the internal capacity for both identifying and changing gender inequalities. Coleman and Ripping (2000) describe two types of collaborators that they formed different kinds of partnerships with. The first type are the “internal partners” that the researchers started negotiating with and that acted as gatekeepers in setting up the details of the project. The second type is the “work groups,” consisting of people that were part of the project because the project targeted their work setting. For the researchers it was important to generate a deeper commitment for the project from these groups. In relation to both these types of partners the collaborations should establish trust and allow them to be active partners, as this is of essence for a gender equality change project.

Feminist theory as activism means keeping in mind, while doing research and writing theory, also focusing explicit attention to how this can contribute to informing and transforming society (Risman 2004). Risman (2004) emphasizes the importance for critical feminist scholars to ask what mechanisms construct gender inequality and how these can be transformed in order to create a more just world. Hence, feminist researchers must “seek to understand how and why gender gets done, consciously or not, to help those who hope to stop doing it” (Risman 2004, 445). She also emphasizes that although organizational rules and institutional laws have by now been rewritten to be gender-neutral, gender inequality persists. It is therefore important to focus the social change agenda within feminism on the interactional and cultural dimension of gender.

Gender activists need to understand the mechanisms of when and how inequality is constructed and reproduced in order to be able to develop strategies that can challenge and change it (Risman 2004). One such strategy involves consciously disrupting interactive processes, status expectations and cognitive bias in our immediate social setting that re-create hierarchies in everyday life. This type of disruption can for example be done through not accepting or adapting to a subordinate position.

### Implementation Theory

This study adopts a theoretical framework which combines the action research approach with implementation theory to systematize, conceptualize and theorize micro change agents practices.

Implementation research is: “the scientific study of methods to promote the systematic uptake of research findings and other evidence-based practices into routine practices” (Eccles and Mittman 2006, 1). Implementation involves efforts, activities, actions and practices carried out to put programs or plans into practice to accomplish a formal, clearly identified goal (Fixsen et al., 2005). Typically, implementation is understood through a model which distinguishes between different stages in a policy cycle where implementation is preceded by agenda-setting, policy formulation and decision-making and followed by evaluation and termination (Jann and Wegrich 2007). The implementation stage is often a long process, often spanning during several years, can involve complex processes and is ideally constituted of at least the following so-called core elements: specification of details regarding the execution, allocation of resources (budget, personnel etc.), and decisions about practices and activities to be executed (Jann and Wegrich 2007).

Implementation is a complex process where decisions are executed and activities initiated under varying conditions (Carey et al., 2019). It is also a critical process as execution is often changed, distorted, delayed or blocked (Jann and Wegrich 2007). Implementation is thus associated with several problems for example due to the complexity of operations in the particular implementation context, where different and sometimes contradictory values and goals need to be considered (Eccles and Mittman 2006). It has, however, also emerged that some of the problems encountered in the specific implementation settings can be removed by collaborations between researchers and stakeholders, professionals, users and/or decision-makers (Carey et al., 2019).

## Methodology, Method and Empirical Data

This article draws on empirical data produced during a 4-year action research project, which targeted gender inequalities in science and research, and involved partners in eight European countries and ten RPOs. In seven of the ten RPOs tailor-made GEPs were implemented during the 4 years of the project (2013–2016). The overall purpose of these GEPs was to contribute to closing the gender gap in science and research. The seven tailor-made GEPs were developed within the structural change project by the micro change agents (and formally accepted and adopted by the seven RPOs). The GEPs contained a total of over 100 different gender equality measures that targeted a range of organizational and administrative processes and procedures and aimed for example to create gender-sensitive recruitment, retention and promotion policies, support and improve work-life balance, establish a more inclusive work culture and raise awareness of gender issues in the RPOs. In this article, however, the specific measures implemented are not the focus of the analysis. Instead the intention is to explore the experiences of the change agents as they implemented the GEPs.

The seven GEPs were implemented by around 20 micro change agents; the number and the individuals involved in the project changed slightly over the 4 years. The reason for this was due to professional or private changes of the people involved, or contractual exits. The micro change agents involved in the implementation of the GEPs acted officially for a period of 4 years and within the context of the time limited project duration. The majority of the change agents were women and their biological age differed from their late 20ties to late 60ties. Their academic age also differed, meaning that their experience of science, research and teaching varied. The role, position and status of the micro change agents varied between the RPOs. Both educators, administrators, and practitioners were involved. Some of the micro change agents were professors with long experience as teachers and researchers, others had more precarious positions with time-limited contracts which would terminate at the end of the project. The academic background of the change agents was also diverse. While the majority of the change agents were active within social sciences, the humanities, engineering and technology were also represented. The background and theoretical and practical knowledge within the field of gender equality change and the experience of previous change projects also varied greatly between the micro change agents, with some not having any previous experience while others possessed several years of previous involvement of practical gender equality work or theoretical knowledge of feminist theory and a gender perspective. The group of micro change agents was thus characterized by great heterogeneity, which was an advantage in the project, as everyone could contribute with their specific viewpoints and perspectives. More detailed information on the micro change agents will not be provided here due to confidentiality and research ethical considerations.

The empirical data analyzed in this article was produced by these micro change agents as they reported and reflected on the implementation process. The data was collected by the authors of the article during these 4 years using monitoring tools, developed by the authors^1^, facilitating a longitudinal observation of micro change agency and change practices in different change settings (c.f., e.g., Dawson 2019).

The implementation of the GEPs and the impact of the GEPs was tracked by monitoring activities throughout the project. Monitoring was characterized by a mixed-method approach and the monitoring tools collected both qualitative and quantitative monitoring data, to ensure that reliable and nuanced information was collected (cf. Lipinsky and Schäfer 2016). The analysis in this article draws on the empirical data collected by means of three qualitative monitoring tools, which were developed based on an ethnographic approach (cf. Lincoln and Guba 1985). Using qualitative methods when monitoring implementation processes produces rich data that reveal whether the implementation is progressing satisfactorily or if some corrective measures are necessary (cf. Chen 1990; Patton 2011). Adopting an interpretative, realist and dialogue approach to monitoring, the qualitative monitoring tools were also designed to provide the micro change agents with space for personal and collective reflections and for exchanges of experiences between them during the implementation phases (cf. Coleman and Ripping 2000; Pawson and Tilley 2004).

These monitoring tools prompted the micro change agents to submit written reports and reflections, using templates specifically tailored for each monitoring tool, and with various designs including questions, tables and mind-maps to be used to structure their accounts and narratives. The written reports and reflections were sometimes produced by the micro change agents individually and sometimes collectively during discussions and workshops. The discussions and workshops were organized during project meetings while most of the individual reports and reflections were collected between these meetings.

The first of the qualitative monitoring tools used to produce the data that this article draws on is the *Self-Assessment of Change Agent Role Monitoring Tool* which was especially developed to stimulate the self-reflection of the micro change agents by asking them to describe their personal experiences of implementing GEPs. This tool documented success factors for implementation and challenges, especially resistance, focusing on access to, and lack of, different kinds of resources important for efficient implementation. The template for the tool consisted of six questions and was designed to leave generous space to elaborate replies. The questions were simple but yet considered to be the most relevant to collect data about micro change agents’ experiences of factors that strengthened and hindered the execution of change practices, for example: What could strengthen you in your role as change agent for gender equality in your RPO? The second qualitative monitoring tool, developed within the project and used in this article, was the *Most Significant Change Technique Monitoring Tool*, inspired by the most significant change methodology (Dart and Davies 2003), intended to collect information about different types of changes. Similarly, the third monitoring tool, the *Incremental Transformation Monitoring Tool* was inspired by a theoretical model, more specifically a model for organizational change, created by John P. Kotter (1995), to systematize the different practices involved in successful organizational change (cf. Chen 1990). The monitoring tools were primarily designed to capture the micro change agents own experiences and the primary function of the tools, and the templates used to collect the experiences, was to stimulate reflections, discussions and knowledge exchange.

## Analysis and Results

The findings in this article illustrate the multifaceted character of micro change agency for gender equality, focusing on six different change practices reported on by the micro change agents. However, it is important to emphasize that the presentation of the practices as six distinctive categories is solely the result of the authors’ thematic analysis of the data in order to systematize the complex and challenging work performed by the micro change agents.

### Practice 1. Communication

One of the key change practices reported on by the micro change agents in this study was also identified as essential for macro change agents by Kelan and Wratil (2018): communicating. Communication practices were described having several different dimensions, three of the most central of these communication dimensions involved *what* to communicate, *with whom* to communicate it and *how* to communicate it. The latter aspect was expressed by a micro change agent, emphasizing the importance of “using the right language” depending on the communication partners. Another micro change agent expressed the necessary aspects of successful and efficient change agency: “knowing who to talk to and how; knowing which arguments have to be used with whom etc.”

The micro change agents identified and emphasized the importance of communicating and disseminating the identified need for change, the vision for change and why change was necessary. Staying in touch about how and why change is needed with colleagues and supervisors is a constant process that is a significant part of change agency and influences and impacts more or less all other change practices. If communication practices are not included in implementation processes the desired and expected progress might soon come to a halt, thus one micro change recommended to “maintain awareness by continually raising the issues in open forums” In his developed 8-step model for change (Kotter, 1996), Kotter also emphasizes the importance of regular, easily understandable and open communication within the change process in order to involve as many organizational members as possible in it and at the same time give them room for concern.

Regarding the dimension *who* to communicate with, the micro change agents expressed and emphasized the need to target stakeholders. Key actors are essential to target with communication practices in order to receive resources and gain legitimacy in the organization, necessary for the change attempts (see below). One of the micro change agents expressed these practices as a recommendation for an efficient micro change agency: “Spend time talking to and securing support of senior colleagues.” Previous research has also highlighted the importance of communication practices to persuade senior managers and leaders to support change projects such as the implementation of GEPs (cf. Bustelo 2003).

The micro change agents described the dimension of *how* to communicate as distinctly different from the communication practices that are important for macro change agents for gender equality (Kelan and Wratil 2018). For CEOs and senior leaders, communication is rather uncomplicated, involving explaining the so-called business case for gender equality and expressing organizational and personal commitment to fairness in career prospects. In contrast, for micro change agents, communication is not always as forthright. Previous research has highlighted the so-called “policy of persuasion,” meaning that the success of gender equality actions depends on a personal factor (Bustelo 2003, 391). For macro change agents this personal factor is manifested in the communication of organizational and personal commitment to gender equality goals. Micro change agents need to use more elaborate strategies to persuade stakeholders to support these actions and these strategies tend to be informal and personal, for example being patient, avoiding confrontation or using a sense of humor (Bustelo 2003).

The micro change agents in this study expressed how change practices used for communication also involved using different *means* for communication: social networks, intranet as a publishing platform, newsletters and even the creation of specific meetings, referred to as “open spaces” to discuss the implementation and present ideas for change. One of the micro change agents described the important aim of these communication practices as: “selling the idea”.

Communication practices were also mentioned as contributing to building communities for change and building trust and legitimacy, presented below as separate change practices.

### Practice 2. Community-Building

The second practice, identified in the empirical data as an essential practice for micro change agents, involved building a community of committed and engaged colleagues, co-workers and organizational leaders in order to mobilize both stakeholders and change “targets” (i.e. those who the change practices target) in their organization. The micro change agents community-building practices thus targeted both the two groups which Coleman and Ripping (2000) emphasize as important. Previous research has also emphasized the need to recruit so-called “allies” in the organization (cf. e.g., Eriksson-Zetterquist & Renemark 2016). One of the goals of these community-building practices was to build commitment to change goals among organizational members. To build such communities the communication practices (see above) were essential. But communicating was not enough, also other resources and strategies were necessary for the communities to be established and enduring. And several different reasons were identified for why community-building was so essential for micro change agents. Several of them emphasized the importance of: “Having contacts, knowing the right people.” This often referred to gate-keepers, stakeholders and decision-makers: “I am now free to contact the Rectorate [i.e., Vice-Chancellor] directly”.

For macro change agents (who often themselves belong to the groups of gate-keepers, stakeholders and decision-makers) the equivalent practice, as identified by Kelan and Wratil (2018), is building ownership. This was also acknowledged by one of the micro change agents who emphasized the importance of spending time on: “trying to engage people in the organization to take ownership of the actions planned, as there was possibly too much for one person to drive forward.” Community-building could thus be a necessary practice in order to cope with an extensive change project.

Accordingly, one of the most conducive factors for a micro change agent was to have other, more formal roles in the department, institution, or workplace in addition to the change agent role, a role that tends to be informal in organizations such as these. Having a formal role in HR or as staff council, for example, was an advantage because it provided a platform to build a network within the organization or to use already existing professional relationships for the cause. In addition, such a position can also facilitate access to information, which in itself can be challenging for individuals who are hired, for example, only for the duration of the project and are unfamiliar with organizational structures and the prevailing culture. For the latter group, it is essential that they network internally or that they can draw on the network resources and contacts of senior staff members. One of the micro change agents explained this aspect of the community-building practices: “I was proactive in talking to and maintaining relationships with people in the organization, which facilitated successful action plan implementation.” An additional recommendation for community-building by one change agent was pursuing an interdisciplinary approach by involving people from different fields and status groups, to benefit from their specific institutional insights.

Another important aspect in relation to the practice of community building and networking with like-minded people is the mutual empowerment and collective processing of setbacks, which can occur directly, for example, by actively blocking equality policy measures, or in a more subtle, indirect way, such as through information gatekeeping (c. Husu 2004). One of the micro change agents exclaimed: “I see this project as an empowering activity.” Community-building was thus not only a practice of direct use for implementing change. It also had a more indirect purpose of providing motivation and support for the micro change agents, especially, but not exclusively, in critical situations (see practice 6 below). In addition to establishing and maintaining relationships within the organization, networking with other micro change agents from similar institutional settings was therefore very important to many of the micro change agents. This exchange helps to reflect on one’s own experiences and at the same time supports mutual learning, be it formal, through information on successfully implemented gender equality measures, or informal, through reports on individual actions with resistance and possible ways to counter them. Specific networks for female researchers and feminist institutional networks were also mentioned as helpful and supportive by two individuals.

### Practice 3. Building Trust and Legitimacy

While macro change agents can usually legitimize and account their commitment to structural change by virtue of their professional role and the associated hierarchies, this is more difficult for change agents below management level. One of the most notable differences, however, is that macro change agents also cite external pressures as the reason for their commitment to gender equality policy (Kelan and Wratil 2018), while micro change agents in our case do so in part to an intention of social justice or personal experiences of injustice (see practice 6 below for more information). But the micro change agents also mentioned other manners of building trust and legitimacy for their cause.

Outside acknowledgment, in this case, in the form of public funding to implement gender equality plans, helps legitimize the commitment and work of micro change agents inside the RPOs. And the new EU policy of mandatory GEPs for public institutions, which apply for funding, even further underlines this legitimacy. One micro change agent highlighted that receiving funding for a gender equality change project challenged the research imperative that what was announced in the proposal is correctly executed, and thus it legitimized to address the issue of gender inequities at the organizational level in the first place in some of the cases presented. Furthermore, external funding was seen as an important signal within the institution to show that research funding organizations, in this case the EU Commission, are committed to advancing gender equality in science and research institutions: “Getting third-party funding for a project dealing solely with gender equality matters is a good sign for people inside the institution.” And that this signal can help to sensitize some of the colleagues and change targets in the RPOs to be more open to the topic. Apart from this, there is of course also the possibility of a contrary defensive reaction, for example, by colleagues questioning why “such topics” are funded publicly at all.

Another essential point to support the legitimacy of micro change agency are national or regional policies. The micro change agents described how they could use reference to these policies to strengthen their position and have their voices heard:

“Change Agency needs to have funds for implementing positive actions for equal opportunities, needs to have the possibility to counter this mechanism and if necessary to utilize some laws that foresee some kinds of penalty for the institutions. To measure gender equality policies through gender equality indicators is an important step to gain this … not only words!”

The legitimizing effect of policies and regulations was especially experienced by those micro change agents who had a more precarious position in the organization. They particularly found that policies invoked their existence to implement change within the institution and some of them described a new law on gender equality in their country as a window of opportunity for them to increase their efforts and have greater impact with their micro change agency. As reported, policies are thus an important argumentation aid, especially if their non-fulfillment is accompanied by possible sanctions (of a monetary nature). The policies and the sanctions were however something that varied between the different country contexts.

The majority of the micro change agents in the seven RPOs lacked the authority and legitimacy in the organization needed to initiate and stabilize change practices. Instead they used other strategies to compensate for this lack. An important practice described was therefore to form alliances with senior managers (Head of Department, Vice-Chancellors, etc.) who could support and act as sponsors for the change project, thereby increasing the commitment to change in the RPO. The importance of ensuring a top-level support was taken as self-evident by the micro change agents: “top-level executive support is crucial” and as already described change practice 1 (see above) involved communicating with senior colleagues in order to secure their support. A micro change agent emphasized the importance of this to gain trust for the change efforts in the RPO:

“Their buy-in is important to ensure effective implementation on a number of levels. […] Their involvement will encourage other staff to participate or help out and their endorsement will provide legitimacy to the project”.

In the present project, this alliance forming practice (cf. Eriksson-Zetterquist & Renemark 2016) was formalized and institutionalized by introducing the position of the so-called transfer agent, persons from higher management levels who supported the core project staff responsible for the operational project activities in each implementing institution (Thaler 2016). These transfer agents (TAs), who could also be categorized as macro change agents, functioned as an extended branch of the project who could, due to their position and/or seniority, facilitate the change agent’s access to data and at the same time act as ambassadors of the project goals within their institutional network. During the implementation phase, and also looking back at the end of the project, the micro change agents reported how beneficial they found the interaction and alliances with their respective TAs.

### Practice 4. Accumulating and Using Resources

The ability to use various forms of resources is essential for change agents. But before they can be used, they must be accumulated and mobilized. This can be a dilemma especially for those among micro change agents who do not hold a formal position dedicated to organizational change issues, for example, as Equality or Diversity Officer of their RPO, which is connected to dedicated resources. For other organizational members change agency can be regarded as a kind of honorary work. In this case, the advocates for gender equality obtained their own resources by participating in a successful EU application, which resulted in the corresponding funding. This may be a rather unusual practice, and one that is also not often available, as funding policy calls of this nature are rare. Funding is however only the means to gain what is the primary resource necessary for a change project: time. As a non-monetary resource, time, gained through project embedding, was a main focus in the micro change agents’ narratives. Through the official allocation of working hours, which are accounted for within the project, change agents can act without neglecting their actual work. Without project funding, this is problematic and means permanently weighing up how much advocacy work is possible.

In addition to finding their own time resources, change agents are also dependent on the time generosity of colleagues. This was significant for the change agents interviewed in different project phases, something that the micro change agents were aware and grateful of: “We are indebted to all the persons who gave us their time.” For example, at the beginning of the project, when interviews and focus group discussions were conducted to identify staff needs, or when it was a matter of motivating colleagues to participate in an online survey aimed at providing information about the current status of gender equality at the institution. As the project progressed, resources such as access to information by colleagues or supervisors became necessary, as did support from administrative staff not directly associated with the project team who provided input on the collection of personnel data for employment analysis. It was however also noted that colleagues not always could contribute with their time: “Although staff members have been generous with their time, there is still a lack of resources (as time has to be cross-funded with other projects).” This meant that the micro change agents had to be creative with how to accumulate and transfer knowledge in the organization.

To have the resources of others made available for change agency highlights the importance of the three change practices listed above as they were described as vital in securing these resources. One of the micro change agents expressed how accumulating resources was linked to for example change practices related to the communicating practices and the community-building practices which aimed at securing commitment and support: “According to me, changes in the mentality are prerequisite to make any gender measure possible and to obtain a specific budget for the implementation of gender actions.” In order to contribute to a possible change in awareness of others pro gender equality, besides excellent communication skills of micro change agents, a sound expert knowledge is necessary to be able to educate uninformed people in a fact-based manner and thus contribute to their capacity building. Practice 4 is thus closely interlinked with also the next practice of using and transferring knowledge.

### Practice 5. Using and Transferring Knowledge

The use of knowledge as a practice is closely related to the two practices of building trust and legitimacy and communication. Knowledge can be distinguished in two ways here, firstly into evidence-based gender expertise, and secondly into organization-based knowledge. Change agents committed to gender equality often face critics whose attitudes are based on everyday gender knowledge, which they then use as a basis for argumentation to undermine institutional gender equality efforts or to declare them unnecessary. Therefore, it is of high importance that change agents have gender equality knowledge relevant to their institution (Dahmen-Adkins et al., 2019) in order to be able to address specifics, be it by implementing practical measures as well as by presenting and interpreting organizational facts, such as gender disaggregated data on different career stages, decision making boards, pay gaps etcetera. This change practice; using knowledge, thus involved a wide variety of sub practices for the micro change agents, for example collecting and analyzing organizational data and presenting it together with the change visions for organizational members and change targets. In contrast to the practices of macro change agents, micro change agents in most cases have proven gender equality knowledge, while macro change agents invoke the knowledge of experts and practitioners who initiate change by proxy.

Some actors reported that in their work as change agents they can draw on results from previous gender-related research projects or practical consultancy work, which is a valuable knowledge resource for them. The majority of the change agents interviewed were also gender scholars, an aspect that should not come as a surprise, since the consortium’s gender expertise also had to be demonstrated in the course of the project application. This type of knowledge was thus a requirement for joining the project. Some of those acting as micro change agents in this project had also actively advocated for gender equality within their institutions before and after the finalization of the project.

The other type of knowledge, organization-based knowledge, is necessary not only in order to initially produce a GEP, tailor made to address the specific issues at hand in the organization. Knowledge about organizational structures, cultures, traditions etcetera, is essential during different implementation phases, for example in order to deal with resistance. It is also a necessary prerequisite for strategically developing the four previous change practices, described above. Contrary to the gender expertise, not all micro change agents possessed this knowledge at the start of the project, sometimes due to lack of transparency in the organization, but reported on how they acquired it during the duration of it. Access to important information was also provided by other organizational actors. One micro change agent here referred to one more reason to ally with senior colleagues, because: “they know a lot about how the organization works.” This type of knowledge is particularly important for micro change agents for gender equality addressing institutional structures and aiming at structural change in RPOs, rather than targeting individual women through for example training efforts (the so called “fix the women” approach’, cf. Ely and Meyerson 2000).

Knowledge transfer and making relevant gender equality knowledge accessible to people within the institution also represents an important component in ensuring the sustainability of gender equality measures. Only if the rationale behind the introduction of intervention measures or changes in institutional policies is understood, there is a likelihood of acceptance and, in the best case, support.

### Practice 6. Drawing on Personal Motivation

Similar to previous studies (cf. e.g., Parsons and Priola 2013) on change agency, it was also found among the micro change agents involved in the project that a crucial indicator of their activist engagement was rooted in their own experiences of discrimination or exclusion: “There is a personal aspect: as a woman I experienced discrimination and sexism etc. and I see the value of a gender equal and inclusive working culture, so I am very much personally involved.” In this case, being affected by discrimination or exclusion leads to the desire to change one’s own situation, but also that of people in similar situations of inequality, and thus to contribute to an improvement of the working environment. Another micro change agent expressed it similarly: “I can use my personal experience for the process.” Kelan and Wratil (2018) found similar statements among the macro change agents they interviewed. Some of their interviewees also based their commitment to gender equality and/or an inclusive work environment on their own experiences or those of family members.

Drawing on personal motivation can be regarded as a decisive impetus for change agency, even if it does not by definition reflect any concrete action. In addition to the above-mentioned personal experiences as a motivation to work for equality, the general personal commitment to social justice and against inequalities of all kinds, regardless of gender, was also mentioned as an incentivizing motive:

“But then I am also a social justice advocate (…) and I hate unfair situations/conditions. E.g. when people say it is all about performance, everybody can achieve the same things they just have to perform etc. and then decisions are made in favor of persons who did not perform better, but talked at the right time to the right people, then I am alarmed.”

This intrinsic drive for change agency commitment can be particularly helpful when facing setbacks or when critical dissenting voices are raised. Being persistent and unafraid were mentioned as important characteristics by almost all micro change agents. One of them also emphasized the need to keep up the motivation, even when facing obstacles: “Do not get discouraged!”. Therefore, personal motivation, and having strategies for keeping that motivation up also when confronted with resistance and challenges, can be seen as a passive practice whose existence sustains the active action of micro change agents. The personal motivation was fueled by both positive and negative emotions and experiences. The negative experiences that fueled motivation were related to unfair treatment and inequalities as described above, while the positive experiences were based on visible progress and achieved change, even if only small wins (Benschop and Van den Brink 2014).

The perception of positive changes, on an individual as well as on an institutional level, was an important factor that helped the change agents involved to maintain their motivation, as this represents direct and indirect feedback on their own change agency work. On an individual level, these include gaining knowledge about gender equality policy (national, local and organizational), or empowerment of one’s own professional role, partly linked to new work tasks and content: “Management board includes me in many more issues because I could help improve certain topics with my gender knowledge, which is valued”. The observed institutional changes were related to cultural aspects, such as increased gender awareness or transparency, to aspects regarding adapted policies and practices, such as the introduction of a new (gender equal and fair) salary scheme or the implementation of new career indicators, and finally to structural aspects, such as the establishment of a stakeholder network for gender issues and new processes of cooperation: “For example, in the university a cross-process has started at the various administrative services, shared by the governing bodies, for the preparation of Gender Budgeting.”

One last motivating aspect which was mentioned was having an intellectual interest in tackling inequalities, especially in combination with a project which follows an action research approach, which highlights in this case a fluidity between the change agent role and the role of a researcher.

## Discussion and Conclusions

The EU Commission’s recent announcement of a mandatory requirement for institutional GEPs for applicants for EU funding in Horizon Europe underlines the need to focus on change agency and change agents’ scope for action on different organizational levels. Because of this new regulation, especially in institutions that have not yet officially dedicated themselves to institutional gender equality work, individuals will be assigned with this kind of organizational care work, which might not have been the core of their professional life so far. This is a scenario which suggests a future wave of new micro change agents. As reported above, national policies were described by the involved micro change agents as an important asset for establishing legitimacy in relation to their GE change agency work. An aspect that should not be underestimated in this context is the existence or non-existence of national resource centers. In countries without a corresponding GE policy, support mechanisms for change agents are lacking. These can be consulting centers for gender equality issues, national contact points, or (in-)formal networks for the exchange of knowledge and experience. This illustrates that besides increasing the legitimacy of organizational change measures, policies are also directly related on an individual level to the presented practices of community-building, and using and transferring knowledge.

Our analysis of the practices of micro change agents shows clear differences between these and the practices of macro change agents described in the literature. The two groups differ significantly in their access to individual and organizational resources. However, although macro change agents formally possess great organizational resources and micro change agents usually have to adopt a range of different change practices to compensate for a lack of such resources, micro change agents can also draw on personal resources which facilitate change practices and implementation, for example personal motivation.

One issue that challenged our micro agents across institutions, is the fact that RPOs, and universities in particular, have one structural level which determines institutional practices, but slightly different subject-specific cultural levels, which in turn have formal and informal rules and peculiarities. Thus, when talking about the importance of knowledge about one’s own institution, it is beneficial in certain contexts to break this down to departmental or faculty level as well, which emphasizes the importance of Coleman and Ripping’s (2000) collaborative action research approach. Efficiency of change agency can benefit from including institutional members of different fields, all genders and different organizational levels, by making use of their specific formal and tacit knowledge.

Furthermore, we have shown that besides practices with an active character, a practice has emerged that can rather be classified as passive: drawing on personal motivation. In order to persistently maintain this motivation, especially in situations of setbacks or experiences of resistance, micro change agents need to actively develop a certain degree of resilience. Disrupting means questioning existing traditional structures, challenging embedded processes and identifying (hidden) mechanisms of exclusion and inequality. Science and research, and particularly academia, are highly competitive environments where advocacy for change and equality will not be received positively by all organizational members. All involved change agents experienced drawbacks during the project duration to different extents regarding for example lack of resources (primarily time and funding) and explicit and implicit resistance. Managing negative experiences and emotions, such as resistance to implementation measures, as a change agent in a professional organizational context requires learning new strategies for dealing with them, or resorting to tried and tested strategies. The enormous importance of exchange with like-minded people was stressed by all interviewees independently of each other, be it with fellow change agents or allies within the organization, or within national/international networks, and in this case also especially with colleagues within the project consortium. Even though the respective institutional, national and cultural backgrounds of the consortium members were partly very diverse, the exchange on a meta-level about potential strategies to overcome resistance and to reflect on obstacles within specially established monitoring sessions was perceived as highly beneficial.

In addition to the previously mentioned development of resilience to cope with critical situations, micro change agents should also practice self-care. Although this was not explicitly expressed by the interviewees during the monitoring process, it became clear in informal exchanges and talks. Besides personal self-care, this also concerns the individual institutional well-being of micro change agents and their continuing professional career. This aspect is particularly crucial for micro change agents with temporary contracts in change projects, who find themselves in a dilemma: On the one hand, they need to raise their voice to advance the issue of gender equality; on the other hand, it is a rather uncomfortable topic for many institutions, especially for actors in the system who fear losing or sharing some of their privileges. Thus, by advocating the issue, temporary change agents increase their own visibility within the institution, which can lead to positive effects, but also can reduce their chances of getting follow-up assignments. A consequence actually observed or feared by some of the change agents involved.

The importance of self-care and also resilience for micro change agents is an aspect that has been neglected in previous research on this topic. Micro changers often represent a vulnerable group, who need a high degree of resilience: may it be because of their insecure employment conditions, their low degree of power and influence in the organization, or simply because they make themselves visible through their activism for injustice and thus offer a potential target for attack. Related to this is the duty of care of supervisors and macro change agents, who should be aware of the precarious situation micro change agents can find themselves in and not expose them to support their agenda.

The discussion of the results therefore emphasizes the importance of adopting an individual monitoring approach in these projects, to complement the organizational focus, to support and encourage these micro change agents. In order to actively disrupt the existing system, the change agents included in this paper made use of different practices, which were similar to, but yet distinctly different from, macro change agents’ practices.

Finally, we want to emphasize that the use of the different qualitative monitoring tools, developed within the project to provide the micro change agents with possibilities for individual and shared reflections, also facilitated several of the micro change agent practices. Most notably the monitoring tools helped the micro change agents to share stories to keep the motivation up (practice 6) and build a community together (practice 2).

## Data Availability

The datasets presented in this article are not readily available because they contain confidential identifiable data. Requests to access the datasets should be directed to JD-A, jdahmen@soziologie.rwth-aachen.de

## Footnotes

1 Further information on the applied monitoring approach within the project framework can be retrieved in Peterson and Dahmen (2018).
